# A fully automated social interaction chamber for studying social threat learning in mice

**DOI:** 10.3389/fnbeh.2024.1481935

**Published:** 2024-11-05

**Authors:** Ellora M. McTaggart, Noah W. Miller, Maria M. Ortiz-Juza, Nicolas C. Pégard, Jose Rodriguez-Romaguera

**Affiliations:** ^1^Department of Applied Physical Sciences, University of North Carolina at Chapel Hill, Chapel Hill, NC, United States; ^2^Department of Psychiatry, University of North Carolina at Chapel Hill, Chapel Hill, NC, United States; ^3^Department of Biomedical Engineering, University of North Carolina at Chapel Hill, Chapel Hill, NC, United States; ^4^Neuroscience Curriculum, University of North Carolina at Chapel Hill, Chapel Hill, NC, United States; ^5^Neuroscience Center, University of North Carolina at Chapel Hill, Chapel Hill, NC, United States; ^6^Carolina Stress Initiative, University of North Carolina at Chapel Hill, Chapel Hill, NC, United States; ^7^Carolina Institute for Development Disorders, University of North Carolina at Chapel Hill, Chapel Hill, NC, United States; ^8^Department of Cell Biology and Physiology, University of North Carolina at Chapel Hill, Chapel Hill, NC, United States

**Keywords:** threat learning, social fear conditioning, behavior tracking, memory, open-source

## Abstract

Social interactions are fundamental for our survival as a predominately social species. We need and seek positive social interactions to navigate the world. However, when social interactions are negative, and occur in the presence of an aversive event, learning occurs to associate such social interactions as threatening. Gaining insight into the neural circuits that drive social threat learning is crucial for understanding social interactions. Animal models can be leveraged to employ technologies that allow us to track neuronal processes with very high resolution to obtain a better understanding of the neural circuits involved. To accomplish this, we need robust behavioral models that are replicable and high throughput. Here, we present an open-source social interaction chamber that detects social interaction and automatically pairs it with foot shock. The social interaction chamber is designed to easily integrate into modular chambers commonly used for auditory and context threat learning. It contains an array of optical gates that precisely track mouse-to-mouse interactions in real time with digital triggers that can communicate with external devices to deliver a foot shock. We find that pairing social interactions with electric foot shock using our fully automated social interaction chamber is optimal for social threat associations. We further demonstrate that timing of social contact with foot shock produces optimal learning.

## Introduction

Survival requires the continuous regulation of behavioral responses to social stimuli. Past social experiences play a crucial role in shaping future social interactions, thereby enhancing survival. Social experiences contribute to the formation of memories that allow individuals to categorize future social encounters as either safe or threatening (Olsson and Phelps, 2007). These adaptive responses can be categorized into bimodal socially motivated behavioral responses, defined by either approach toward a safe social stimulus or avoidance of a social threat (Nikitin and Freund, 2010; Padilla-Coreano et al., 2022). Understanding the intricate nature of social behaviors is vital for identifying maladaptive behaviors associated with psychiatric disorders linked to social deficits, such as social anxiety, autism spectrum, and post-traumatic stress disorder (in cases where the aversive association is related to a social stimulus). Rodent models can be employed to leverage technological advances that allow us to dissect these maladaptive behaviors and underlying neural circuits with very high resolution (Calhoon and Tye, 2015; Rodriguez-Romaguera et al., 2020).

To understand the formation and recall of social threat memories, research groups have started to employ tasks in rodents that pair social contact with an aversive foot shock (Toth et al., 2012; Zoicas et al., 2014; Xu et al., 2019). One advantage of this type of task is the similarity with auditory threat learning tasks used for decades in neuroscience research (also referred to as auditory fear conditioning) (LeDoux, 2014). This allows us to leverage what we have learned from auditory threat learning tasks in rodents to understand the neural circuits that differ when an aversive outcome is paired with a social stimulus. To facilitate the study of social threat learning and memory, we developed an automated social interaction chamber that precisely measures social proximity between two mice. Custom circuitry in the chamber employs photobeam interruption measurements across an array of infrared photobeams to track the proximity of the experimental and stimulus mice in real time. Photobeam monitoring provides a non-intrusive and highly sensitive means of activity detection in rodents (Nadler et al., 2004). The social interaction chamber is open-source, and features customizable, real-time output capabilities to trigger external systems such as a foot shock for social threat learning applications.

Using the automated social interaction chamber, we found that precise pairing of social contact with a foot shock allows for optimal learning in a social threat learning task. To accomplish this, we performed a three-group design. One group that was allowed to freely interact with a social stimulus and never received a foot shock, a second group that received a foot shock when they were not making social contact with a social stimulus, and a third group that received a foot shock after making social contact with a social stimulus. After 24 h, all mice were tested for social threat memory in a three-chamber social assay. We found that the group in which social contact and foot shock were precisely paired using the social interaction chamber had a significant social aversion in comparison to the other two groups.

## Materials and methods

### Animals

Mice used to evaluate social threat learning were experimentally naive, 8–12 week old adult male and female C57BL6/J mice. Mice used as the social stimulus were unrelated, age-, and sex-matched C57BL6/J mice. 24 h prior to behavioral testing, mice were single-housed and kept isolated throughout behavioral testing. Mice were housed under a reverse light cycle with *ad libitum* access to food and water. Experiments were performed during the dark cycle. All procedures were conducted in accordance with the Guide of the Care and Use of Laboratory Animals, as adopted by the National Institutes of Health, and with the approval of the Institutional Animal Care and Use Committee from the University of North Carolina at Chapel Hill.

### Automated social interaction chamber

**FIGURE 1:**
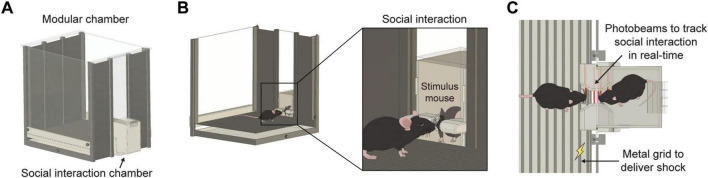
Schematics of an automated social interaction chamber. **(A)** Schematic of behavioral chamber classically used in fear conditioning with side panel replaced with social interaction chamber. **(B)** Schematic depicting social interaction among experimental and social stimulus mice. **(C)** Schematic depicting an aerial view of social contact. The top arrow indicates the placement of an infrared photodiode used to precisely track social interaction in real-time. The bottom arrow indicates a metal grid that will be triggered by a social interaction system to deliver a shock to the experimental mouse upon social contact.

To automatically detect social interactions during social threat learning experiments, we developed an open-source, inexpensive device that is designed for installation into modular test chambers classically used in auditory threat learning experiments (ENV-007-VP, Med Associates, VT, USA) (Figures 1A, B). The social stimulus chamber is equipped with an array of four infrared (IR) photobeam sensors aligned to rapidly detect when physical contact occurs between experimental and stimulus mice (Figure 1C). A custom printed circuit board (PCB) integrated with a microcontroller (Seeeduino XIAO, Seeed Studio, CA, USA) tracks these events and outputs Transistor-Transistor Logic (TTL) states that trigger external systems with TTL input compatibility. In this study, we connected the digital outputs of the automated social interaction chamber to an ANY-maze video tracking system (Version 7.4, Stoelting Co., IL, USA). ANY-maze was programmed to deliver a foot shock to an experimental mouse after precisely 2 s of direct contact with a social stimulus. Below, we provide an outline of a step-by-step guide to building the social interaction chamber system. The digital design files for the fabrication of custom-made parts, the code to program the microcontroller, and additional instructions are available on a dedicated GitHub repository for this project: https://github.com/UNC-optics/Social-Interaction-Chamber.

### Social stimulus chamber assembly

The social interaction chamber was designed to easily slide into one of the side panels of a modular test chamber and to be large enough to allow an adult stimulus mouse to move around comfortably. The chamber measures 55 mm × 60 mm × 93 mm. It is composed of clear and white cast acrylic sheets (Houston Acrylic, TX, USA) that were laser cut (VLS 4.75 laser cutter, Universal Laser Systems Inc., AZ, USA) and bonded using acrylic solvent (IPS16 Acrylic Plastic Cement, WELD-ON Adhesives Inc., CA, USA). The side, back, and bottom walls were constructed (laser cut) from white acrylic sheets (3.18 mm) to maintain high contrast between the white chamber and the dark social stimulus. The partition separating mice was made (laser cut) with clear acrylic sheets (1.59 mm) and contains a 10 mm × 15 mm window for the exchange of sensory cues and to allow physical contact (nose touch) between mice. The top lid is also made with clear cast acrylic and is secured with a hinge to ease the transfer of social stimuli between experiments and to prevent mice from escaping. Both the lid and the entire partition that separates the chambers were made clear to allow cameras placed above the modular chamber to visualize stimulus mice inside the attached social interaction chamber.

### Electronic components assembly

**FIGURE 2:**
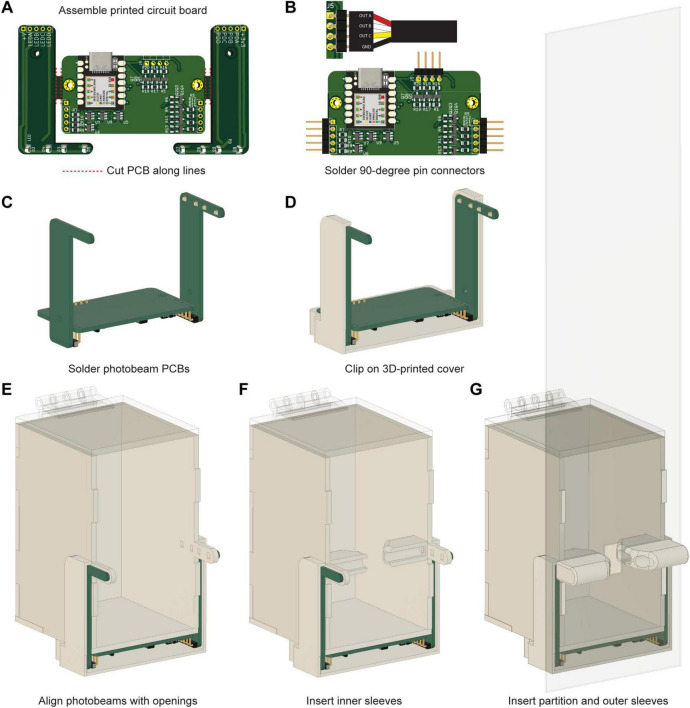
Assembly and integration of electronic circuitry for the social interaction chamber to automate precise detection of social contact. **(A)** Pre-assembled custom-made printed circuit board (PCB) with surface-mounted components. **(B)** PCB 90-degree pin placement and output cable pinout. The J5 connector features ground and three outputs. **(C)** Photobeam arm installation and orientation. The chamber is used as a template for spacing. **(D)** 3D-printed case for PCB protection. **(E)** Alignment of the PCB around the chamber. The four LEDs and four photodiodes must be aligned with the apertures on the acrylic chamber. The cavity between the PCB and case is filled with 2-part epoxy to protect the circuitry. **(F)** Placement of inner diode sleeves. **(G)** Insertion of the transparent partition and outer diode sleeves.

We developed a custom PCB that incorporates an Arduino-compatible microcontroller (Seeeduino XIAO, Seeed Studio, TX, USA) that was used to control all electrical components of the automated social interaction system and to output three TTL signals to trigger external devices. The PCB fabrication and assembly were outsourced (JLCPCB, HK, CN) using the fabrication and assembly files linked in the GitHub with specifications of a 1.6 mm two-layer PCB (no special instructions). The PCB was specifically designed to surround the social interaction chamber. After fabrication, a tampered cutter tool (CHP-170-A Micro Cutter, HAKKO, CA, USA) was used to detach the board into two L-shaped boards with the photobeams, and a rectangular base board with circuitry to drive the LEDs and amplify the photodiode signals (Figure 2A). 90-degree pin headers were hand soldered onto the base board (Figure 2B) and the photobeam boards were subsequently soldered to the opposite end of the pins to secure them to the base in a perpendicular orientation (Figure 2C). Following PCB assembly, an insulated cable (1 m 24AWG 4-Conductor Cable, Tensility International Corp., OR, USA) was hand soldered to a female 4-pin connector (2.54 mm Socket Header, Würth Elektronik, NH, DE) according to the pinout of the PCB outputs (Figure 2B). The exposed wire was covered with heat shrink (Heat Shrink Tubing Kit, 3M, MN, USA) and the cable was set aside for future attachment of the device to external systems.

### Final construction

To prevent damage during experiments, the PCB was protected with a 3D printed external case (MK4S 3D printer, Prusa Research, HMP, CZ) made with white PLA filament (Prusament PLA Pristine White, Prusa Research, HMP, CZ) (Figure 2D). The PCB was fit around the constructed social stimulus chamber and the cavity at the base was filled with epoxy (2-part epoxy, Gorilla Glue, OH, USA) (Figure 2E). Light-directing diode sleeves were also 3D printed (MK4S 3D printer, Prusa Research, HMP, CZ) with white PLA filament (Prusament PLA Pristine White, Prusa Research, HMP, CZ) and were secured over the photobeam light paths with super glue before and after the insertion of the partition (Ultra Gel Super Glue, Loctite Corp., CT, USA) (Figures 2F, G).

### Software installation and programming

To setup the social interaction device, we connected the Seeeduino XIAO to a computer via a USB port. We used Arduino IDE (Version 2.3.2) to compile and upload the code. The Seeeduino hardware definition package was added into the Arduino board manager to manage the microcontroller (instructions).^1^ The programming code, written in C language, was then compiled and uploaded to the board. The program can be described as follows: First, an initial baseline lighting measurement is determined upon calibration (power-on) with no obstructions. The signals from the array of photodiodes are then repeatedly captured and compared to the baseline measurement. Signal differentiation allows for precise location tracking of both mice, as specific patterns of IR photobeam interruptions deduce experimental and stimulus mouse position. The programming script can be customized with up to three outputs indicating specific beam interruption patterns. By default, the device outputs binary serial monitor data as TTL pulses when the stimulus mouse breaks both beams (Inside - “I”), when the experimental mouse breaks both beams (Outside - “O”), or when the experimental mouse is approaching and breaks only one beam (Near - “N”). To ensure accurate detection of “I” and “O” states, the setup requires the mice to simultaneously break both beams. The window size has been optimized to allow for precise nose pokes while preventing the mice from extending into the opposite side, reducing the risk of false triggers. Additionally, each “N,” “I,” or “O” state is streamed through the USB port whenever a status change occurs. After programming, we tested the device by viewing the Arduino serial monitor and assessing live beam break status (0 - no break, 1 - beam broken) and outputs (I, O, N) while blocking the photodiodes using a solid object. We then verified that each output state corresponded to a change of output value in each of the TTL output pins.

On the same computer, ANY-maze software (Version: 7.4) was installed and connected to the ANY-maze digital interface (60064, Stoelting Co., IL, USA). The previously constructed cable was then plugged into the social interaction chamber and the opposite ends of the cable were connected to the terminals of the ANY-maze digital interface. All output wires were attached to the first pin of three separate ports and the ground wire was attached to the second pin of any of the utilized ports. The digital interface configuration settings were then set to TTL inputs and 0V output power for all ports. Power was supplied to the Seeeduino from the computer via a USB-C cable and port status was viewed in the ANY-maze I/O digital interface. Any inconsistencies were resolved by plugging and unplugging the USB-C power source and ensuring that the device underwent calibration in consistent experimental lighting conditions.

### Social threat learning

To test whether the social interaction chamber can be used for social threat learning, we employed a previously described social threat learning paradigm (Toth et al., 2012; Xu et al., 2019) using modular testing chambers (ENV-007, Med Associates, VT, USA). The modular chambers consisted of a transparent polycarbonate box (53.35 × 34.93 × 27.31 cm) equipped with a stainless-steel grid floor (ENV-005A, Med Associates, VT, USA) and removable side panels. The middle panel on one side of the conditioning chamber was replaced with the automated social interaction device (Figure 1A). During experiments, the grid floor was connected to an animal shocker device (SDI Programmable Animal Shocker, San Diego Instruments, CA, USA) that was configured to deliver a shock when triggered by the social interaction device. The modular chamber was enclosed in a sound-attenuated behavior box equipped with a red LED strip to illuminate the box’s interior. A video camera (DMK-22BUC03, The Imaging Source, NC, USA) was positioned above the conditioning chamber to monitor mouse behavior using ANY-maze software.

Twenty-four hours prior to social threat learning, social stimulus mice were habituated to the social interaction chamber for 10 min. For social threat learning, experimental mice were placed into the conditioning chamber and given a 10-min adaptation period prior to introduction of the social stimulus. After this period, an unfamiliar, same-sex social stimulus mouse was placed into the social interaction chamber, and social contact was monitored. One group of mice interacted with the social stimulus for 10 min and never received a foot shock (No shock). A second group was given a 1 s electric foot shock (0.3 mA, pulsed current) when they were not making social contact with the social stimulus (Unpaired social-shock). To ensure that the shocks were unpaired, 1 s foot shocks were triggered by an observer when the mouse was in the arena that was on the opposite side of the social stimulus. A third group received a foot shock during social contact with the social stimulus (Paired social-shock). Social contact was defined by all photobeams breaking for a continuous period of 2 s. The time for shock delivery for the Unpaired social-shock group was matched to what naturally occurred in the Paired social-shock group (∼2 min into the session). At the end of each session, experimental and social stimulus mice were removed, and the chamber was thoroughly cleaned with a disinfectant (Roccal-D Plus, Zoetis, Inc., NJ, USA).

### Social threat memory

To investigate whether mice demonstrate social threat learning, we assessed social behaviors across experimental mouse groups using a three-chamber social assay (61 cm × 30.5 cm × 30.5 cm), as previously described (Moy et al., 2004). 24 h after social threat learning, experimental mice were placed in the middle chamber of a large three-chamber arena and allowed to explore for 10 min. After the acclimation period, the same social stimulus mouse used for social threat learning was placed into a small chamber and positioned in the corner of one of the two outer chambers. An empty small chamber was used as a non-social stimulus and placed in the corner of the opposite outer chamber. A video camera (DMK-22BUC03, The Imaging Source, NC, USA) was positioned above the three-chamber arena and ANY-maze software was used to analyze mouse behavior and time spent in the social and non-social zones during a 10-min testing period. Zones for analysis were defined by a square arena in front of either the social chamber (Social zone) or the empty chamber on the opposite side (Non-social zone). At the end of testing, the experimental and social stimulus mice were removed. The large three-chamber arena and the two small chambers were wiped thoroughly with a disinfectant (Roccal-D Plus, Zoetis, Inc., NJ, USA) to remove odors.

### Statistics

Statistical analyses were performed using GraphPad Prism (version 10.2.2, La Jolla, CA, USA). Sample sizes were ∼6–8 mice per group. One outlier from the unpaired social-shock group was excluded in the analyses of social interaction behavior in the three-chamber social assay due to exhibiting zero exploratory behavior. Equality of variances was tested using the Brown-Forsythe test and normality of datasets was tested using Shapiro-Wilk’s test. If variances between groups are equal, and data followed a normal distribution, comparisons were tested using one-way analysis of variance (ANOVA) followed by Tukey’s *post hoc* test. Otherwise, if variances were not equal, and data followed a normal distribution, Brown-Forsythe ANOVA followed by Dunnett’s T3 multiple comparison test was used. Statistically significant differences are marked as *p < 0.05, **p < 0.01, ***p < 0.001, ****p < 0.0001.

## Results

We provide instructions for the construction of a social interaction chamber that integrates with systems commonly used in auditory threat learning experiments (Figures 1A, B). This chamber includes an array of photobeams for precise detection of social contact between two mice (Figure 1C). It is powered by a custom printed circuit board and features an Arduino-compatible microcontroller that can be programmed with up to three custom TTL outputs based on specific photobeam break patterns (Figure 2B). This setup ensures high temporal resolution across all electrical components and facilitates the precise control of external systems by generating output signals to activate various devices. The physical design incorporates reproducible laser-cut acrylic structures and 3D-printed casing components to preserve longevity of device (Figure 2G). The selection of white and transparent materials provides a consistent white environment, which supports optimal behavioral tracking in experiments with low light conditions.

**FIGURE 3:**
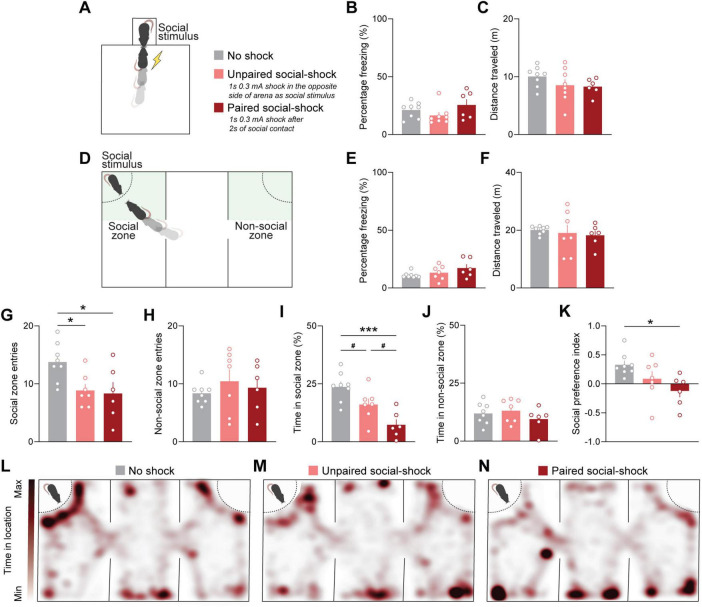
Automated pairing of social contact with foot shock induces social threat learning. **(A)** Schematic representing experiment protocol to assess social threat learning using an automated social interaction chamber. **(B)** Percent time freezing was not different across groups during social threat learning [one-way analysis of variance (ANOVA); F_2_,_19_ = 1.751, *p* = 0.2005]. **(C)** Distance traveled was not different across groups during social threat learning [one-way (ANOVA); F_2_,_19_ = 1.398, *p* = 0.2714]. **(D)** Schematic of three-chamber social assay used to test recognition of social threat 24-h after learning. Green rectangles indicate zones defined in ANY-maze software as social and non-social for mouse behavioral analysis. **(E)** Percent time freezing was not significantly different across groups in the three-chamber social assay [one-way analysis of variance (ANOVA); F_2_,_18_ = 1.940, *p* = 0.172]. **(F)** Total distance traveled was not significantly different across groups in the three-chamber social assay (Brown-Forsythe ANOVA; F_2_,_9_._496_ = 0.2495, *p* = 0.7841). **(G)** One-way ANOVA indicates significant difference between both the groups × entries into the social chamber (F_2_,_18_ = [4.977], *p* = 0.0190). Tukey’s *post hoc* test indicated that reduced entries into the social zone were exhibited in the Unpaired social-shock group compared to the No shock group (adjusted *p* = 0.0453) and Paired social-shock group compared to No shock group (adjusted *p* = 0.0329). No significant differences in number of social zone entries were found in unpaired social-shock vs. social- paired social-shock groups (adjusted *p* = 0.9637). **(H)** Number of non-social zone entries was not significantly different between groups [one-way ANOVA; F_2_,_18_ = 0.4938, *p* = 0.6183). **(I)** One-way ANOVA indicates significant difference between the groups × percent time in social zone (F_2_,_18_ = [11.61], *p* = 0.0006). Tukey’s *post hoc* test indicated the paired social-shock group exhibited reduced time spent in social zone compared to the No shock group (adjusted *p* = 0.0004). Statistical trends were observed between the Unpaired social-shock group compared to the No shock group (adjusted *p* = 0.0748) and the Unpaired social-shock group compared to the Paired social-shock group (adjusted *p* = 0.0563). **(J)** Time spent in the non-social zone was not significantly different across groups (one-way ANOVA; F_2_,_18_ = 0.9227, *p* = 0.4151). **(K)** Social preference index was calculated by (Time in social zone – Time in non-social zone)/(Time in social zone + Time in non-social zone). One-way ANOVA analysis demonstrated a difference between groups × social preference index (F_2_,_18_ = [4.508], *p* = 0.0259). Tukey’s *post hoc* test indicated a significant difference in social preference index in Paired social-shock group vs. No shock group (adjusted *p* = 0.0210). No statistical differences were observed with Unpaired social-shock group vs Paired social-shock group and No shock group (adjusted *p* = 0.2368 and 0.4033, respectively). **(L–N)** Heatmaps of grouped data depicting average time spent throughout locations in the three-chamber social assay. Data are shown as mean ± SEM. Sample size is ∼6–8 mice per group. *p < 0.05, ***p < 0.001, ^#^*p* = 0.05–0.1 (trend).

To test the capabilities of the social interaction chamber to automate social threat learning, we configured the output signals of the social interaction device to precisely control ANY-maze hardware to trigger a 1 s foot shock upon social contact between experimental and social stimulus mice. Groups consisted of mice that did not receive a shock upon social contact (No shock), mice that did receive a shock but only when located on the opposite side of the social stimulus (Unpaired social-shock), and mice that received a shock that was precisely triggered upon social contact (Paired social-shock) (Figure 3A). After 10 min of exposure to these three conditions, mice were returned to their individual home cage. Paired social-shock mice received between 1 and 2 shocks on average, depending on the number of times the mice performed 2 s of continuous social interaction over the test duration. The number and timing of shocks was matched between the Paired social-shock and Unpaired social-shock conditions. To assess if shocked mice exhibited locomotion differences compared to No shock mice, we analyzed freezing and total distance traveled. Statistically significant differences were not observed between any of the groups (Figures 3B, C).

To assess whether mice display social threat memory, we exposed mice to the three-chamber social assay 24 h after social threat learning. Mice were placed in a three-chamber arena in which one chamber contained the same social stimulus presented during social threat learning (Figure 3D). ANY-maze video tracking software was used to automate the collection of mouse movements across user-defined social and non-social zones. Freezing behavior and total distance traveled between groups were not statistically different (Figures 3E, F), indicating that sociability metrics are not dependent on locomotor activity. To assess differences in social approach behavior, we compared the number of entries into social zones across groups. Both the Unpaired social-shock and Paired social-shock groups entered the Social zone fewer times compared to the No shock group (Figure 3G). No difference in entries to the Non-social zone across groups was found (Figure 3H). Although both the Unpaired and Paired social-shock groups exhibited a decrease in number of entries to the social chamber, only the Paired social-shock group spent significantly less time in the social zone (Figure 3I), as compared to the non-social zone (Figure 3J). This difference was also reflected when calculating the social preference index, in which the Paired social-shock group was the only one to show a significant reduction in social preference compared to the No shock group (Figure 3K). These differences can also be observed in heat maps depicting the average amount of time spent across all mice within a group in the large three-chamber arena. Notably, the Paired social-shock group spent more time in the locations at the opposite end of the social stimulus (Figures 3L–N). Taken together, these findings highlight the importance of precise triggering of shock upon social contact to induce social threat learning.

## Discussion

Classic auditory and context-based threat learning paradigms are commonly used to elucidate the neural processing of threats (Maren, 2003; Herry et al., 2010; Johansen et al., 2011; Rodriguez-Romaguera and Quirk, 2017; Sangha et al., 2020; de Oliveira Alvares and Do-Monte, 2021; Moscarello and Penzo, 2022; Diehl et al., 2024). These models are associated with specific sensory modalities and their related neural circuits. In contrast, social threat learning integrates multiple sensory modalities including auditory, visual, olfactory, and tactile processing (Padilla-Coreano et al., 2022). This multimodal information processing implies the engagement of discrete neural circuits, each capable of eliciting and modulating unique defensive behaviors, highlighting the need for novel approaches to capture these varied social behaviors.

In this study, we developed a social interaction system for seamless integration into modular behavioral chambers, which are commonly used in fear conditioning experiments. The system has custom circuitry and photobeams designed to detect real-time social interactions between mice. When it detects social contact, it generates binary serial monitor data as TTL pulses, which can be used to trigger external systems. We used this automated system to trigger hardware and deliver a foot shock at the precise moment of social contact. One day later, we assessed social threat learning using the three-chamber social assay. Our results show that pairing social contact with a foot shock using this automated system induced robust social threat learning, as indicated by a reduced number of entries and time spent near the social stimulus in the paired social-shock group. Additionally, pairing social contact with shock induced aversion to the social stimulus, with experimental mice relocating to areas in the three-chamber social assay that were farthest from the social stimulus. Interestingly, we found that mice that received foot shock in the presence of a social stimulus (but outside of social contact) also exhibited social threat learning, although to a lesser degree. This finding highlights the need for temporal gating between social interaction and shock triggering to optimize social threat learning. Further, we would like to highlight that the 0.3 mA shock used in our studies is lower than prior studies that typically use shock intensity levels above 0.6 mA (Toth et al., 2012; Zoicas et al., 2014; Xu et al., 2019). We believe this 0.3 mA level of shock was appropriate to produce social avoidance in the absence of freezing responses, which may be suitable to study more clinically relevant social anxiety-like phenotypes.

Although this automated social interaction system is effective for studying social threat learning, there are limitations to consider. First, consistent lighting conditions must be maintained throughout the experiment, as rapid changes in baseline lighting can affect the device’s ability to detect social contact between mice. The chamber was specifically designed to fit within Med Associates modular test chambers, so the design files will need to be adapted for use with other behavioral chambers or experimental setups. Additionally, while the small window size and diode sleeves are crucial for ensuring device accuracy, they may limit the naturalistic representation of social interactions. Nonetheless, our results show that the social interaction system is sufficient to induce social threat learning across mice.

In this study we demonstrate the capability of the automated social interaction system to detect periods of social interaction and precisely trigger a shock upon social contact. Importantly, this feature can also be used to trigger other devices, such as sounds, lights, reward delivery, lasers for optogenetics, and any other device that can receive a TTL pulse. Additionally, the TTL pulses from this system can serve as time markers for electrophysiological and calcium imaging approaches that monitor neuronal activity dynamics during social interaction. The custom circuitry can be adapted for other novel applications that integrate social interactions into behavioral tasks. For these reasons, this fully automated social interaction chamber offers a convenient and versatile tool for studying socially relevant behaviors, such as social threat responses.

## Data Availability

The raw data supporting the conclusions of this article will be made available by the authors, without undue reservation.

## Footnotes

1
https://wiki.seeedstudio.com/Seeed_Arduino_Boards/
